# A Relação entre PCR e EAC: Associação de Proteína C Reativa para Proporção de Albumina em Pacientes com Ectasia Isolada da Artéria Coronária

**DOI:** 10.36660/abc.20200580

**Published:** 2021-01-27

**Authors:** Iran Castro, Hugo Antonio Fontana

**Affiliations:** 1 Instituto de Cardiologia Porto AlegreRS Brasil Instituto de Cardiologia - Direção científica, Porto Alegre, RS – Brasil

**Keywords:** Doença Arterial Coronária, Dilatação Patológica, Aterosclerose, Lipoproteínas, Estresse Oxidativo, Fatores de Risco

Ectasia arterial coronária (EAC), definida como um aumento do diâmetro coronariano 1,5 vez o diâmetro do leito adjacente normal,^1^ é um achado incomum em cineangiocoronariografias, com incidência de 1,2 a 4,9%.^2^ Na maioria das vezes, está relacionada com doença aterosclerótica coronariana (DAC),^3^ sendo que apresentam vários fatores em comum, como acumulação de lipoproteínas na camada íntima, infiltração de células inflamatórias, ativação do sistema renina-angiotensina e geração de estresse oxidativo, com expansão e remodelamento arterial. Os elevados níveis de oxido nítrico causam vasodilatação e ativação excessiva de metaloproteinases da matriz extracelular, resultando em dilatação vascular.^4^ Pode ainda, menos comumente, estar relacionada com doença de Kawasaky, doenças do tecido conjuntivo, infecciosas ou autoimunes.

A incidência é maior em homens, hipertensos e tabagistas. Usuários de cocaína apresentam maior incidência de EAC e aneurismas coronarianos.^5^ Interessantemente, diabetes melito (DM) parece não ter relação com EAC, podendo até mesmo ser fator protetor, fato relacionado com a inibição da expressão das metaloproteinases da matriz extracelular.^6^

A elevação da proteína C reativa (PCR) é fator amplamente relacionado com aumento da atividade inflamatória e do risco cardiovascular,^7 - 8^ assim como a redução dos níveis séricos de albumina (A).^9^

Em recente publicação^10^ com 102 pacientes com EAC e o mesmo número de participantes sem EAC, os autores demonstraram que pacientes com EAC apresentaram alta relação PCR/albumina (CAR) em comparação ao grupo-controle, levando à possibilidade de identificação de EAC e sua relação inflamatória, implicando prognóstico e manejo terapêutico. Este estudo é pioneiro em mostrar tal associação e certamente ajudará na prática cardiológica. No entanto, diferenciar se os níveis elevados desta relação estão relacionados a ectasia coronariana ou aos fatores de risco mais prevalentes no grupo de casos, como tabagismo, hipertensão e dislipidemia, e o consequente aumento da prevalência de DAC, ainda carece de estudos prospectivos ou que, talvez, usem como grupo controle pacientes com DAC e sem ectasia coronariana.
