# Supplementary material for: A Relação entre PCR e EAC: Associação de Proteína C Reativa para Proporção de Albumina em Pacientes com Ectasia Isolada da Artéria Coronária
Source: Arq Bras Cardiol. 2021 Jan 27;116(1):55. [Article in Portuguese] doi: 10.36660/abc.20200580 (PMC8159518; doi:10.36660/abc.20200580)
Supplement: Translation

# Translation: The Relationship between CAR and CAE: Association of C-Reactive Protein to Albumin Ratio in Patients with Isolated Coronary Artery Ectasia

**Authors:** Iran Castro, Hugo Antonio Fontana

**Affiliations:** 1 Brazil Instituto de Cardiologia - Scientific direction, Porto Alegre, RS – Brazil

**Keywords:** Coronary Artery Disease, Dilatation, Pathologic, Atherosclerosis, Lipoproteins, Oxidative Stress, Risk Factors

Coronary artery ectasia (CAE), defined as an increase in coronary diameter 1.5 times the diameter of the normal adjacent bed,^1^is an uncommon finding in coronary angiography, with an incidence of 1.2 to 4.9%.^2^Most of the times, it is related to coronary atherosclerotic disease (CAD),^3^and they have several factors in common, such as lipoprotein accumulation f in the intimal layer, inflammatory cell infiltration, activation of the renin-angiotensin system and oxidative stress generation, with arterial expansion and remodeling. The high levels of nitric oxide cause vasodilation and excessive activation of extracellular matrix metalloproteinases, resulting in vascular dilation.^4^Less commonly, it can also be related to Kawasaky disease, connective tissue, infectious or autoimmune diseases.

The incidence is higher in men, hypertensive individuals and smokers. Cocaine users have a higher incidence of CAE and coronary aneurysms.^5^Interestingly, Diabetes Mellitus (DM) seems to be unrelated to CAE, and may even be a protective factor, a fact related to the inhibition of the expression of extracellular matrix metalloproteinases.^6^

The increase in C-reactive protein (CRP) is a factor largely related to increased inflammatory activity and cardiovascular risk,^7 , 8^as well as the reduction in serum albumin levels (A).^9^

In this recent publication^10^with 102 patients with and the same number without CAE, the authors demonstrated that patients with CAE had a high CRP/albumin (CAR) ratio compared to the control group, leading to the possibility of identifying CAE and its inflammatory association, implying prognosis and therapeutic management. This study is a pioneer in showing this association, and will certainly help in cardiology practice; however, to differentiate whether the high levels of this association are related to coronary ectasia or to the most prevalent risk factors in the group of cases, such as smoking, hypertension and dyslipidemia, and the consequent increase in the prevalence of CAD, prospective studies are still necessary, or perhaps, using patients with CAD and without coronary ectasia as a control group.
